# Modulation of Forward Propulsion and Foot Dorsiflexion by Spinal and Muscular Stimulation During Human Stepping

**DOI:** 10.3390/life16020226

**Published:** 2026-01-29

**Authors:** Sergey Ananyev, Ivan Sakun, Vsevolod Lyakhovetskii, Alexander Grishin, Tatiana Moshonkina, Yury Gerasimenko

**Affiliations:** Pavlov Institute of Physiology, Russian Academy of Sciences, 199034 St. Petersburg, Russia; ananevss@infran.ru (S.A.); sakunia@infran.ru (I.S.); lyakhovetskiiva@infran.ru (V.L.); agrishin@infran.ru (A.G.); moshonkina@infran.ru (T.M.)

**Keywords:** transcutaneous spinal cord stimulation, spinal locomotor networks, stepping movements, muscle and posterior roots stimulation

## Abstract

(1) Background: We developed a novel technology that regulates human locomotion using transcutaneous electrical spinal cord stimulation to activate spinal locomotor networks and posterior root stimulation to activate leg flexor and extensor motor pools during swing and stance phases, respectively. This technology effectively restores walking in post-stroke individuals while forward propulsion in the stance phase and foot dorsiflexion in the swing phase are insufficient. In this study the effectiveness of regulating the stance and swing phases while healthy volunteers walked on a treadmill with transcutaneous electrical stimulation of the posterior roots, leg muscles, and their combined effects has been examined. (2) Methods: We analyzed the kinematic characteristics of stepping movements in healthy participants with spinal stimulation of the posterior roots and flexor/extensor leg muscles. (3) Results: Our findings clearly show that posterior root stimulation at T12 combined with tibialis anterior muscle stimulation during the swing phase effectively regulates foot dorsiflexion, whereas posterior root stimulation at L2 combined with hamstrings and medial gastrocnemius stimulation during the stance phase effectively regulates forward propulsion. (4) Conclusions: Combined stimulation in the stance and swing phases within the same gait cycle resulted in the most coordinated stepping, and effective control of forward propulsion and foot dorsiflexion.

## 1. Introduction

According to the World Health Organization, between 200,000 and 500,000 new cases of traumatic spinal cord injury (SCI) are reported worldwide each year [1]. Despite significant advances in clinical care, current therapeutic approaches for spinal cord injury remain limited in their ability to restore lost functions of the central nervous system [2,3]. Consequently, the development of effective treatment strategies capable of alleviating the functional consequences of SCI has become a major focus of contemporary neurological research.

One promising approach is electrical stimulation of the spinal cord, which has been shown to activate spinal neural networks known as locomotor pattern generators [4]. Activation of these networks can induce walking-like movements in humans [5,6]. However, locomotor patterns elicited by spinal cord stimulation alone are generally insufficient to produce fully functional locomotion and primarily serve as a trigger for movement initiation. For instance, in both horizontal and vertical body positions, thoracolumbar spinal cord stimulation induces rhythmic lower-limb movements predominantly at the hip joint, with preferential activation of proximal leg muscles [7].

Successful locomotor behavior is believed to require effective sensorimotor integration, including descending supraspinal commands, activation of spinal locomotor networks, and afferent feedback from the limbs. Accordingly, neurorehabilitation protocols for patients with motor impairments of various etiologies often combine spinal cord stimulation with locomotor training [8,9,10]. A major achievement of this approach has been the demonstration of independent overground walking in individuals with SCI under spinal cord stimulation, assisted by external support devices such as canes or walkers to maintain balance [11,12].

Nevertheless, it has been observed that during electrically assisted walking in individuals with SCI, stimulation targeting the region of spinal locomotor networks does not sufficiently activate the muscles of the lower leg [13,14]. As a result, these muscles are unable to generate adequate propulsive force during the stance phase or to produce effective dorsiflexion of the foot during the swing phase. This limitation has been partially addressed through the use of non-invasive multimodal spinal cord stimulation, which combines continuous activation of spinal locomotor networks with phase-specific stimulation of posterior roots to selectively engage flexor and extensor motor pools during distinct phases of the gait cycle [15].

Using this stimulation paradigm, we observed effective forward propulsion during the stance phase and robust foot dorsiflexion during the swing phase in healthy volunteers. However, in individuals with SCI, these effects were less pronounced, likely due to structural and functional alterations in the affected muscles [16,17].

The aim of the present study was to evaluate the effectiveness of modulating the stance and swing phases of gait using transcutaneous electrical stimulation of the posterior roots, lower-limb muscles, and their combined application during treadmill walking in healthy volunteers.

## 2. Materials and Methods

### 2.1. Participants and Ethics

This study was approved by the Ethics Committee of the Pavlov Institute of Physiology (Minutes No. 25-01, 25 February 2025). All participants provided written informed consent. The study was conducted in accordance with the Declaration of Helsinki.

Sixteen healthy volunteers (13 males and 3 females; age range: 19–35 years) with no history of neuromusculoskeletal disorders participated in the study. Participants walked on an Ortorent treadmill (Moscow, Russia) at a constant speed of 2 km/h. This velocity was adjusted to match the gait velocity following a stroke [18,19].

### 2.2. Protocol

The study included seven consecutive experimental conditions: walking without stimulation and walking with transcutaneous spatiotemporal stimulation applied to the posterior spinal roots and lower-leg muscles of the right limb. The initial condition consisted of walking without stimulation (Table 1).

### 2.3. Transcutaneous Spinal–Muscular Stimulation

**Figure 1:**
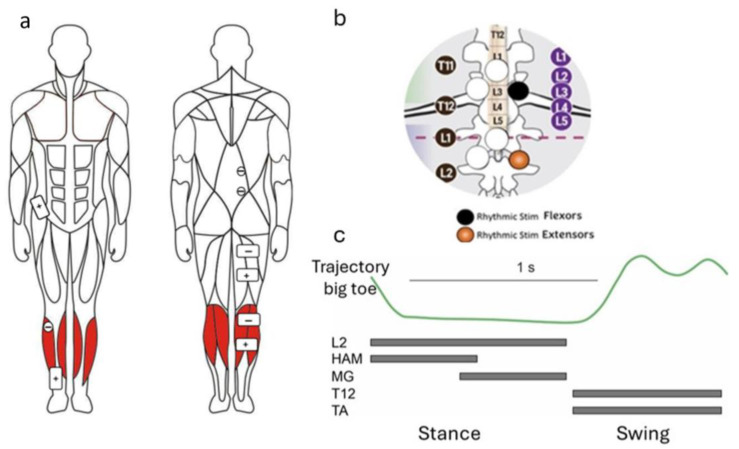
Location of electrodes for stimulation of flexor and extensor leg muscles (**a**) and for stimulation of posterior roots, by [15] (**b**). (**c**) shows the experimental paradigm stimulation of posterior roots (L2) and extensor leg muscles (hamstring (HAM) and m. gastrocnemius (MG) during stance phase as well as stimulation of posterior roots (T12) and flexor leg muscle (m. tibialis anterior (TA)) during swing phase. The lateral electrode at T12 delivers rhythmic stimulation at 30 Hz during the swing phase, while the lateral electrode at L2 delivers rhythmic stimulation at 15 Hz during the stance phase. Black circles indicate vertebra numbers, white rectangles denote spinal cord segments, violet circles mark the levels of spinal cord segments, and the red dotted line shows the end of the lumbar region of the spinal cord.

To stimulate the posterior spinal roots, electrodes were placed on the skin at the T12 and L2 vertebral levels. Round cathode electrodes (diameter 2.5 cm; ValuTrode^®^, Axelgaard Manufacturing Co., Fallbrook, CA, USA) were positioned laterally, approximately 1.5–2 cm from the spinal midline (Figure 1). A common rectangular anode electrode (5 cm × 10 cm; ValuTrode^®^, Axelgaard Manufacturing Co., Fallbrook, CA, USA) was placed over the right iliac crest.

For stimulation of the right tibialis anterior muscle, a round cathode electrode (diameter 3.2 cm; ValuTrode^®^, Axelgaard Manufacturing Co., Fallbrook, CA, USA) was positioned over the belly of the muscle below the lateral condyle of the tibia, while a rectangular anode electrode (5 cm × 10 cm) was placed on the lower third of the muscle above the myotendinous junction (Figure 1).

To stimulate the gastrocnemius muscle (MG), the cathode electrode was placed on the upper portion of the muscle between its medial and lateral heads, while the anode electrode was placed distally. For stimulation of the hamstring muscles (HAM), the cathode electrode was positioned on the proximal posterior thigh beneath the gluteal fold, and the anode electrode was placed distally at mid-thigh. Rectangular electrodes (5 cm × 10 cm; ValuTrode^®^, Axelgaard Manufacturing Co., Fallbrook, CA, USA) were used.

Transcutaneous spinal–muscular stimulation was delivered using monophasic pulses at 30 Hz for T12 stimulation and 15 Hz for L2 stimulation. Pulse duration was 1 ms with a 5 kHz carrier frequency. It has been reported that epidural stimulation with frequency 30 Hz at T12 most effectively modulates flexor motor pools, whereas stimulation at 15 Hz induces coactivation of antagonist muscles and promotes sustained limb extension when delivered to L2 in individuals with SCI [13,20]. These effects were subsequently demonstrated using transcutaneous spinal cord stimulation in individuals with SCI [21,22,23]. Consistent findings have also been reported in healthy subjects, demonstrating frequency-dependent modulation of gait kinematics during transspinal stimulation [24]

Lower-limb muscles were stimulated at 35 Hz using pulses of 0.3 ms duration with a 5 kHz carrier frequency. Stimulation was delivered using a Neuroprosthesis-NP stimulator (Cosyma LLC, Moscow, Russia). Stimulation intensity was individually adjusted in a seated position. For posterior root stimulation, intensity was set to elicit paresthesia without discomfort. For muscle stimulation, intensity was adjusted to produce sufficient plantarflexion and dorsiflexion without discomfort [25,26].

An algorithm previously developed for controlling posterior root stimulation [15] was applied. Phase detection was performed using inertial sensors incorporating a gyroscope and accelerometer, placed on the anterior thigh above the right knee. During walking, the sagittal plane angle of the sensor relative to the vertical axis was monitored. The onset of hip flexion determined the beginning of the swing phase. The gait cycle was defined as the interval from heel strike to the subsequent heel strike of the same limb [27,28].

During the stance phase, stimulation was applied as follows: L2 roots from 0–55%, HAM from 0–30%, and MG from 25–55% of the gait cycle. During the swing phase, stimulation of T12 posterior roots and the TA muscle was applied from 57–98% of the gait cycle [29] (Figure 1c).

### 2.4. Recording of Kinematic Characteristics of Walking

Three-dimensional marker trajectories were recorded at 100 Hz using a 10-camera optoelectronic motion capture system (Oqus 500, Qualisys, Gothenburg, Sweden). Spherical reflective markers (diameter 19 mm) were used. Calibration errors were <0.5 mm within a working volume of approximately 4 m × 2 m × 2.5 m [30]. Reflective markers placed on the lateral epicondyle of the humerus, greater trochanter, lateral femoral epicondyle, lateral malleolus, heel, and hallux were used to reconstruct lower limb kinematics. Motion reconstruction was performed in Qualisys Track Manager (Qualisys, Gothenburg, Sweden). A predefined marker model was used for biomechanical assessment of lower limb movements [31].

### 2.5. Data Analysis

**Figure 2:**
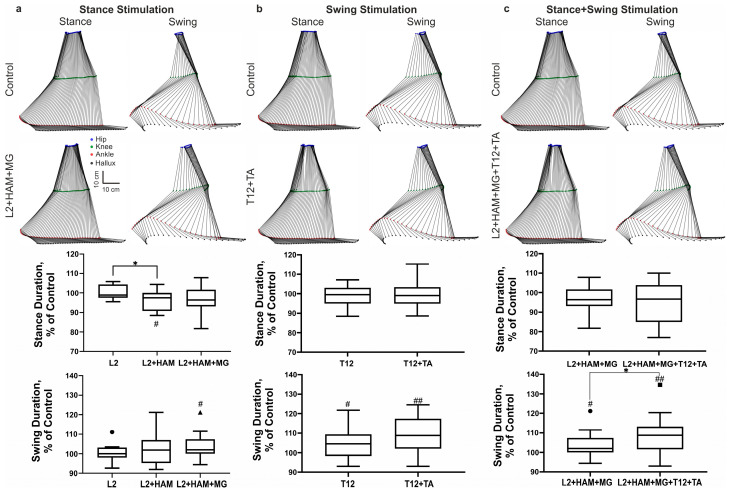
Effects of spinal–muscular stimulation on gait characteristics. (**a**) Stick diagrams of movements in the stance and swing phases and boxplots of the duration of the stance and swing phases during stimulation in the stance phase. (**b**) Stick diagrams of movements and boxplots of the duration of the stance and swing phases during stimulation in the swing phase. (**c**) Stick diagrams of movements and boxplots of the duration of the stance and swing phases during combined stimulation of the stance and swing phases. Note: # *p* < 0.05; ## *p* < 0.01 relative to control values, * *p* < 0.05 relative to stimulation conditions. In the boxplots, the box extends from the first quartile (Q1) to the third quartile (Q3), denoting the interquartile range (IQR), which encompasses the middle 50% of the data, and the horizontal line within the box indicates the median (Q2). The whiskers extend from the edges of the box to the smallest and largest data values, which lie within 1.5 IQR of Q1 and Q3, respectively, reflecting the bulk of the distribution without extreme outliers (marked by dot, square and triangle).

Marker coordinate data were exported to TSV files and processed using an in-house Python-based software (v.3.12) tool. This custom software was used to calculate more than 80 spatiotemporal and kinematic gait parameters. In addition, the marker data were used to visualize the trajectories of the hip, knee, ankle, heel, and hallux throughout the gait cycle. Stick diagrams were generated to illustrate the phases of the right leg step cycle (Figure 2).

For each stimulation condition, parameters obtained from five gait cycles were averaged and normalized to the corresponding parameters from five gait cycles recorded during treadmill walking without stimulation (control) at each experimental stage (Table 1). Data distributions were assessed for normality using the D’Agostino–Pearson test. To determine significant deviations from 100% at the 0.05 significance level, either Student’s *t*-test or Wilcoxon’s test was applied, depending on the distribution. Comparisons between datasets were performed using paired Student’s *t*-test or Wilcoxon’s paired test. When comparing more than two datasets, one-way repeated-measures analysis of variance with Geisser–Greenhouse correction followed by Tukey’s post hoc test, or Friedman’s test followed by Dunn’s multiple comparisons test, was used for normally and non-normally distributed data, respectively.

All analyses were performed using Prism 9.0 (GraphPad Software, La Jolla, CA, USA). Movement parameters are presented as mean ± standard deviation.

## 3. Results

### 3.1. Stimulation of the Posterior Root L2 and Extensor Leg Muscles During the Stance Phase

Figure 2 shows the stick diagrams of reconstructed gait movements when walking on a treadmill belt with spinal and muscular stimulation applied in different phases of the gait cycle. ANOVA had shown the influence of stimulation on the duration of the stance phase (F(1.245, 17.42) = 5.03; η^2^ = 0.52). Post hoc Tukey’s multiple comparisons had shown that the spinal cord stimulation of the posterior root (L2) during the stance phase had no significant effect on the duration of this phase, whereas combined stimulation of the posterior root L2 and muscle stimulation (HAM) significantly shortened the duration of the stance phase—4% relative to L2 stimulation (*p* = 0.0263). L2+HAM+MG stimulation led to a decrease in the stance phase duration as a tendency. At the same time, such stimulation significantly increased the subsequent unstimulated swing phase by +4% (d = 0.62, W(16) = 56; *p* = 0.0269) (Figure 2a).

**Figure 3:**
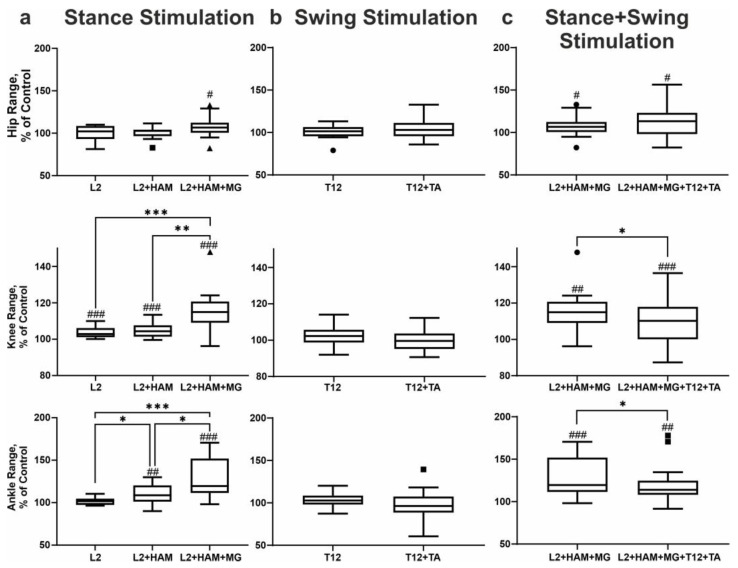
Change in angular displacements (peak to peak) in the hip (top row of boxplots), knee (middle row of boxplots), and ankle angles (bottom row of boxplots) during stimulation of the stance phase (**a**), swing phase (**b**), and combined stimulation of the stance and swing phases (**c**). Note: # *p* < 0.05; ## *p* < 0.01; ### *p* < 0.001 relative to control values, * *p* < 0.05; ** *p* < 0.01; *** *p* < 0.001 relative to other stimulation conditions. In the boxplots, the box extends from the first quartile (Q1) to the third quartile (Q3), denoting the interquartile range (IQR), which encompasses the middle 50% of the data, and the horizontal line within the box indicates the median (Q2). The whiskers extend from the edges of the box to the smallest and largest data values, which lie within 1.5 IQR of Q1 and Q3, respectively, reflecting the bulk of the distribution without extreme outliers (marked by dot, square and triangle).

When L2 was stimulated during the stance phase, the amplitude of movement in the hip and ankle joints remained unchanged, while in the knee joint the amplitude increased by +4% (d = 1.25, t(15) = 4.83, *p* < 0.001). The application of L2+HAM stimulation resulted in no alteration in the range of motion of the hip joint. In the knee joint the range of motion increased by +5% (d = 1.23, t(15) = 4.78, *p* < 0.001), and in the ankle joint by +10% (d = 0.83, t(15) = 3.20, *p* = 0.0065). The application of combined L2+HAM+MG stimulation resulted in a significant augmentation in the amplitude of movement in the hip, knee, and ankle joints by 8% (d = 0.60, t(15) = 2.321, *p* = 0.0359), 16% (d = 1.35, W(16) = 116, *p* < 0.001), and 29% (d = 1.30, t(15) = 5.044, *p* < 0.001), respectively (Figure 3a). The Friedman test has shown that the application of different types of stimulation leads to different changes in range of motion of the knee joint (χ^2^(2) = 17.2, *p* < 0.001, η^2^_Q = 0.28). Dunn’s multiple comparisons test has shown that the range of motion for L2+HAM+MG stimulation is higher than for L2 and L2+HAM stimulation (*p* < 0.001, and *p* = 0.003, respectively). Similarly, ANOVA has shown that the application of different types of stimulation leads to different changes in range of motion of the ankle joint (F(1.267, 17.73) = 16.21, *p* < 0.001, η^2^ = 0.91). Tukey’s post hoc comparisons test has shown that the range of motion for L2+HAM+MG stimulation is higher than for L2 and L2+HAM stimulation (*p* < 0.001, and *p* = 0.0121, respectively); the range of motion for L2+HAM stimulation is higher than for L2 stimulation (*p* = 0.0121). At the same time, during L2+HAM+MG stimulation (that is during stance) the length of the hip joint movement trajectory shortened by −9% (d = 0.81, t(15) = 3.136, *p* = 0.0073), the knee joint shortened by 8%, (d = 0.81, t(15) = 3.15, *p* = 0.0071), and the ankle joint shortened by −4%. Due to the application of L2+HAM+MG stimulation during stance, in the subsequent unstimulated swing phase, the length of the hip joint movement trajectory increased by +13% (d = 0.78, t(15) = 3.04, *p* = 0.0088), the knee joint by +20% (d = 1.48, t(15) = 5.75, *p* < 0.0001), and the ankle joint by +3% (d = 0.76, t(15) = 2.03, *p* = 0.0109) (Figure 3a).

Stimulation of L2, L2+HAM and L2+HAM+MG during stance phase increased the height of the ankle lift by +9% (d = 0.72, W(16) = 80, *p* = 0.0215), +20% (d = 1.50, t(15) = 6.05, *p* < 0.001) and +35% (d = 1.61, t(15) = 6.16, *p* < 0.001), respectively. Conversely, stimulation of L2+HAM+MG during the stance phase did not change the trajectory lengths of the heel or hallux during the stance phase, but increased the heel trajectory length by +6% (d = 1.27, t(15) = 4.91, *p* < 0.001) and the hallux trajectory length by +4% (d = 0.94, t(15) = 3.63, *p* = 0.0028) during the swing phase. Thus, activation of the extensor motor neuron pools (L2 stimulation) in combination with activation of the extensor muscles (HAM+MG) during stance phase shortened the duration of this phase, shortened the trajectories of the hip, knee and ankle joints, and caused the leg to rise above the treadmill belt and perform an intense swing of the limb.

### 3.2. Stimulation of the T12 Posterior Root and Leg Flexor Muscles During the Swing Phase

During the swing phase, stimulation of the T12 posterior roots and combined spinal–muscle stimulation (T12+TA) increased the duration of the swing phase by +4% (d = 0.66, t(15) = 2.64, *p* = 0.0185) and +9% (d = 0.99, t(15) = 3.97, *p* = 0.0012), respectively (see Figure 2b).

Meanwhile, the subsequent unstimulated stance phase remained unchanged.

T12 and T12+TA stimulation did not change the amplitude of movements in the hip, knee and ankle joints (Figure 3b). With T12+TA stimulation during swing the length of the hip joint movement trajectory increased by +13% (d = 0.82, t(15) = 3.17, *p* = 0.0068). The knee joint trajectory increased by +8% (d = 1.32, t(15) = 5.12, *p* < 0.001) and the ankle joint trajectory by +3% (not significant).

When T12+TA was stimulated during the swing phase, the height of the ankle lift increased by +5% (d = 0.52, t(15) = 2.86, *p* = 0.0120). The length of the heel movement trajectory increased by +4% (d = 0.55, t(15) = 2.21, *p* = 0.0427), while the length of the hallux movement trajectory increased by +3% (not significant). In the unstimulated stance phase, the length of the heel and hallux movement trajectories did not change.

T12+TA stimulation in the swing phase led to a change in the difference between the first and second peaks of hallux lift. In the control, the first peak was 16 mm higher than the second one, but with stimulation, this indicator decreased to 6 mm. The change in this indicator characterizes an increase in the height of the maximum rise in the second peak by +39% (d = 0.90, t(15) = 3.61, *p* = 0.0026).

Thus, T12+TA stimulation in the swing phase led to a lengthening of the swing phase, an increase in the length of the trajectory in the hip, knee, and ankle joints, and an increase in the lift and swing of the leg above the treadmill belt, associated with an increase in foot dorsiflexion.

### 3.3. Combined Stimulation of the Posterior Roots of L2 and the Extensor Muscles of the Legs in the Stance Phase and the Posterior Roots of T12 and the Flexor Muscles of the Legs in the Swing Phase

With this stimulation, the duration of the stance phase remained unchanged, while the duration of the swing phase increased by +10% (d = 0.84, t(15) = 3.26, *p* = 0.0056) (see Figure 2c). This increase is also significantly higher than the increase during L2+HAM+MG stimulation (d = 0.53, W(16) = 46, *p* = 0.042).

The application of L2+HAM+MG stimulation leads to a more significant increase in range of motion of the knee and ankle joints relative to combined stimulation (d = 0.53, W(16) = −74, *p* = 0.0353; d = 0.54, W(16) = −72, *p* = 0.0413, respectively). The amplitude of hip joint movements did not change in the stance phase but decreased in the swing phase by −1% (d = 0.58, t(15) = 2.23, *p* = 0.0428). Movement amplitude in the knee joint decreased by −1% (d = 0.83, t(15) = 3.20, *p* = 0.0064) in the stance phase and by −5% (d = 0.97, t(15) = 3.78, *p* = 0.0020) in the swing phase. The amplitude of plantar flexion in the ankle joint increased by +1% (d = 0.60, t(15) = 2.29, *p* = 0.0378), while the amplitude of dorsiflexion in the ankle joint did not change.

The length of the hip joint trajectory decreased insignificantly by −2% in the stance phase, but increased by +27% (d = 0.63, t(15) = 2.42, *p* = 0.0295) in the swing phase. The knee joint trajectory decreased by −2% in the stance phase and increased by +33% (d = 0.87, t(15) = 3.38, *p* = 0.0045) in the swing phase. The length of the ankle joint trajectory decreased by −6% in the stance phase (d = 0.57, t(15) = 2.20, *p* = 0.0451) and increased by +5% in the swing phase.

With combined stimulation, the height of the ankle lift increased significantly by +33% (d = 1.22, t(15) = 4.74, *p* < 0.001). The length of the heel movement trajectory did not change in the stance phase, but increased by +7% (d = 0.69, t(15) = 2.66, *p* = 0.0187) in the swing phase. The length of the hallux movement trajectories in the stance and swing phases remained unchanged. Thus, combined stimulation of L2+HAM+MG during the stance phase, followed by stimulation of T12+TA during the swing phase, did not change the duration of the stance phase but increased the swing phase. Combined stimulation led to slight changes in amplitude at the hip, knee and ankle joints, increased leg lift above the treadmill belt and decreased angular displacement at the hip and ankle joints during the stance phase, while increasing angular displacement at the knee and ankle joints during the swing phase.

### 3.4. Characteristics of Foot Movement During Spinal–Muscular Stimulation

**Figure 4:**
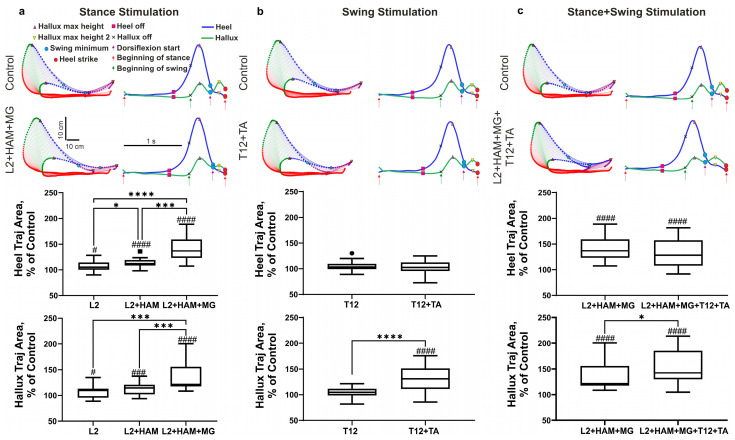
Effects of spinal–muscular stimulation. Trajectories of heel and hallux movement during stance (**a**), swing (**b**) and combined stance + swing (**c**) stimulation. Boxplots of changes in the area within the trajectory (integral) of the heel and hallux for each of the stimulation conditions. Note: # *p* < 0.05; ### *p* < 0.001; #### *p* < 0.0001 relative to control values, * *p* < 0.05; *** *p* < 0.001; **** *p* < 0.0001 relative to other stimulation conditions. In the boxplots, the box extends from the first quartile (Q1) to the third quartile (Q3), denoting the interquartile range (IQR), which encompasses the middle 50% of the data, and the horizontal line within the box indicates the median (Q2). The whiskers extend from the edges of the box to the smallest and largest data values, which lie within 1.5 IQR of Q1 and Q3, respectively, reflecting the bulk of the distribution without extreme outliers (marked by dot and square).

Figure 4 shows the trajectories of the reconstruction of heel and hallux movement during spinal–muscular stimulation. On the stick diagrams, the stance phase is highlighted in red, the beginning of the swing phase (initial swing) is highlighted in green (lift), and the heel/hallux drop (mid swing) is highlighted in blue, and the second lift (start dorsiflexion/terminal swing) is highlighted in purple. On this figure the trajectories of the heel (blue line) and hallux (green line) in the gait cycle, indicating the start of lift (heel/hallux) (heel off), foot lift-off (start of the swing phase), and limb landing (heel strike) is presented. Kinematic analysis shows that, after positioning the limb on the treadmill belt, the heel and hallux remain on the belt for a period of time. At the end of the stance phase, the heel begins to rise while the hallux still touches the belt. Once the heel reaches a certain height, it and the hallux simultaneously lift above the treadmill belt. At this moment, the swing phase begins. The difference in behavior between the heel and hallux is that the heel goes through three stages in the stance phase—contact with the treadmill belt, lifting off above the belt, and heel lift—while the hallux goes through two stages: contact with the belt and lifting above it (Figure 4a).

ANOVA had shown that the stimulation in the stance phase (L2, L2+HAM, L2+HAM+MG) increases the integrals of heel and hallux movement (Figure 4a) (F(1.302, 18.22) = 28.21, η^2^ = 0.92, *p* < 0.0001, and F(1.29, 18.06) = 22.87, η^2^ = 0.86, *p* < 0.0001, respectively). Post hoc Tukey’s multiple comparisons had shown that the maximal increase in these integrals was for L2+HAM+MG stimulation (both +39%) (heel, hallux: *p* < 0.001 both relative to L2 and L2+HAM stimulation).

According to kinematic data, the heel lift in the stance phase does not change during spinal–muscular stimulation (Figure 4a (red color)), and the effect of stimulation appeared in the subsequent swing phase. Stimulation in the stance phase raises the heel above the treadmill belt (swing) significantly higher than in the control. Thus, with L2+HAM+MG stimulation in the stance phase, the maximum heel lift above the treadmill belt increased by +35% (d = 1.61, t(15) = 6.25, *p* < 0.001) relative to the control (Figure 4a). Stimulation in the stance phase also affected the movement characteristics of the hallux in the unstimulated swing phase. With L2 and L2+HAM+MG stimulation in the stance phase after the hallux left above the treadmill belt, the first peak of hallux lift increased by +8% (d = 0.69, W(16) = 80, *p* = 0.0215) and +30% (d = 1.59, t(15) = 6.16, *p* < 0.001), respectively. With L2 stimulation, the second peak of hallux lift increased by +5% (not significant), and with L2+HAM+MG stimulation, the second peak decreased by −15% (d = 0.37, W(16) = −70, *p* = 0.0479).

**Figure 5:**
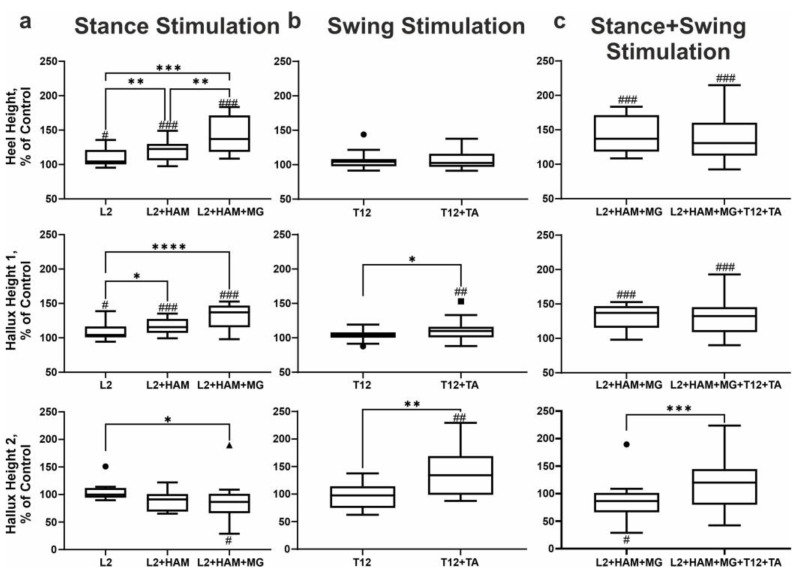
Change in heel lift height (top row of boxplots), hallux 1 (max lift height of first peak of hallux) (middle row of boxplots) and hallux 2 (max lift height of second peak of hallux) (bottom row of boxplots) during stimulation of the stance phase (**a**), swing phase (**b**), and combined stimulation of the stance and swing phases (**c**). Note: # *p* < 0.05; ## *p* < 0.01; ### *p* < 0.001 relative to control values, * *p* < 0.05; ** *p* < 0.01; *** *p* < 0.001; **** *p* < 0.0001 relative to other stimulation conditions. In the boxplots, the box extends from the first quartile (Q1) to the third quartile (Q3), denoting the interquartile range (IQR), which encompasses the middle 50% of the data, and the horizontal line within the box indicates the median (Q2). The whiskers extend from the edges of the box to the smallest and largest data values, which lie within 1.5 IQR of Q1 and Q3, respectively, reflecting the bulk of the distribution without extreme outliers (marked by dot, square and triangle).

When stimulating T12 and T12+TA in the swing phase, the integral of heel movement within the gait cycle did not change, while the integral of hallux movement increased by +33% when stimulating T12+TA (d = 1.33, t(15) = 5.34, *p* < 0.0001) (Figure 4b). The maximum height of the first and second peaks of the hallux lift did not change during T12 stimulation. However, during T12+TA stimulation, the maximum height of the first peak increased by +12% (d = 0.71, W(16) = 100, *p* = 0.0076), and the second peak increased by +39% (d = 0.90, t(15) = 3.61, *p* = 0.0026) (Figure 5b). The maximum height of the first and second peaks of the hallux lift did not change during T12 stimulation. However, during T12+TA stimulation, the maximum height first peak increased by +12% (*p* < 0.01), and the second peak increased by +42% (*p* < 0.01) (Figure 5b).

With combined stimulation of L2+HAM+MG during stance and T12+TA during swing, the integral of heel movement within the gait cycle increased by +33% (d = 1.18, t(15) = 4.59, *p* < 0.001), and the integral of hallux movement increased by +54% (d = 1.60, t(15) = 6.19, *p* < 0.001) (Figure 4c). This increase in the integral of hallux movement is significantly higher than for L2+HAM+MG stimulation (d = 0.60, t(15) = 2.33, *p* = 0.0351).

With L2+HAM+MG+T12+TA stimulation, the height of the first peak of the hallux lift increased by +31% (d = 1.22, t(15) = 4.72, *p* < 0.001), and the second peak increased by +19% (not significant) (Figure 5c).

Thus, L2+HAM+MG stimulation during stance increased the maximum heel lift by +42% (*p* < 0.001), while T12+TA stimulation during swing did not significantly change heel lift height.

## 4. Discussion

At present, two principal spinal neuromodulation strategies aimed at the control of human walking have been described. The first employs tonic epidural stimulation of the spinal cord to modulate and optimize the excitability of spinal neural networks [32]. The second strategy relies on rhythmic stimulation of afferent fibers within the posterior roots that project to specific motor neuron pools, thereby enabling phase-dependent control of the stance and swing phases of stepping [13].

Non-invasive transcutaneous spinal cord stimulation has been developed to regulate stepping movements in both neurologically intact individuals [7] and individuals with spinal cord injury (SCI) [8,33]. More recently, a novel approach to gait modulation was introduced, combining non-invasive electrical stimulation of spinal locomotor networks with phase-specific stimulation of posterior roots to activate flexor and extensor motor pools of the lower limbs during distinct phases of the step cycle [15,34]. Using this approach, we previously demonstrated improved kinematics during voluntary, non-weight-bearing, locomotor-like stepping in a patient with SCI classified as AIS A [35]. However, we also observed that, despite these improvements, this neuromodulation strategy alone did not enable effective body propulsion during the stance phase or adequate foot dorsiflexion during the swing phase in individuals with SCI.

In the present study, we demonstrated that spatiotemporally alternating stimulation of posterior roots projecting to leg flexor and extensor motor pools, combined with direct stimulation of corresponding flexor and extensor leg muscles, produced marked alterations in gait structure (see Video S1). Specifically, stimulation applied during the stance phase enhanced forward propulsion, stimulation during the swing phase induced foot dorsiflexion, and stimulation during both phases modulated both forward propulsion and foot dorsiflexion.

### 4.1. Regulation of Stepping Movements During Combined Spinal and Muscle Stimulation

The results demonstrate the effectiveness of combining transcutaneous stimulation of the L2 posterior roots, which project to extensor motor pools of the leg, with direct stimulation of the r-leg’s extensor muscles (hamstrings, HAM, and medial gastrocnemius, MG) for regulating the stance phase of treadmill walking. Previous work has shown that rhythmic transcutaneous stimulation of the L1 posterior root during the stance phase shortens stance duration, increases hip joint excursion, and reduces movement amplitudes at the knee and ankle joints [8,34]. In contrast, the present study revealed that rhythmic stimulation of L2 combined with HAM and MG during the stance phase tended to shorten stance duration while simultaneously increasing movement amplitudes at the hip, knee, and ankle joints. Moreover, this combined stimulation significantly increased ankle and heel elevation above the treadmill belt (Figure 5).

These findings suggest that the combined stimulation of the L2 posterior root and extensor muscles leads to an interaction between spinal and peripheral inputs that facilitates limb elevation and effective foot movement—effects that are not observed with posterior root stimulation alone.

During treadmill walking, transcutaneous stimulation of posterior roots at the T12 level, which project to leg flexor motor pools, combined with direct stimulation of the tibialis anterior (TA) muscle, effectively modulated the swing phase of gait. Stimulation of the T12 posterior root alone had only a limited effect compared with combined T12+TA stimulation. In both conditions, heel lift height remained unchanged; however, ankle elevation increased with combined T12+TA stimulation. Previous studies have reported that rhythmic stimulation of the T11 posterior root during the swing phase produces only a minor increase in swing duration but enhances flexion amplitudes at the hip and ankle joints, as well as knee lift [8].

In the present study, combined spinal–muscular stimulation (T12+TA) during the swing phase significantly increased swing duration (Figure 2b). Although joint movement amplitudes at the hip, knee, and ankle remained unchanged (Figure 3b), the trajectory lengths of these joints increased. Moshonkina et al. [36] demonstrated that continuous simultaneous stimulation of the spinal cord at C5–C6, T11–T12, and L1–L2 results in a maximum foot lift of 10 ± 2 cm during the gait cycle, whereas rhythmic stimulation of MG and TA during specific gait phases increases foot elevation to 12 ± 2 cm. Notably, this value does not further increase with combined spinal cord and lower-leg muscle stimulation [36].

When stimulation of L2+HAM+MG during the stance phase was followed by stimulation of T12+TA during the swing phase, stance duration remained unchanged, whereas swing duration increased, accompanied by a significant elevation of both the ankle and heel above the treadmill belt. This combined stimulation also increased joint movement amplitudes at the hip, knee, and ankle. Simultaneously, the trajectory lengths of these joints were reduced during the stance phase and prolonged during the swing phase.

Taken together, these results indicate that the combined stimulation of L2+HAM+MG during the stance phase and subsequent stimulation of T12+TA during the swing phase interact synergistically to facilitate forward propulsion and effective foot dorsiflexion during treadmill walking.

### 4.2. Neurophysiological and Biomechanical Correlates of Forward Propulsion and Foot Dorsiflexion During Spinal and Muscular Stimulation

Forward propulsion is a critical component of walking, as it generates the force required to move the body forward. It occurs during the stance phase and is expressed as elevation of the limb above the treadmill belt in combination with active plantar flexion of the foot, resulting in push-off from the support surface. Several key features characterize forward propulsion during combined spinal–muscular stimulation. Rhythmic stimulation of the L2 posterior root during the stance phase increased the integrals of heel and hallux displacement over the gait cycle, as well as the maximum elevation of the ankle, heel, and the first and second peaks of hallux lift. All of these parameters increased by approximately 10% relative to control conditions. When L2 stimulation was combined with stimulation of the extensor muscles (HAM + MG) during the stance phase, these parameters increased by approximately 35–40% relative to control (see Section 3).

These findings indicate that concurrent stimulation of the posterior root and extensor muscles substantially enhances forward propulsion compared with stimulation of the posterior root alone. This observation is consistent with previous reports showing that continuous spinal cord stimulation combined with rhythmic stimulation of the medial gastrocnemius (MG) increases foot lift by approximately 2 cm compared with spinal cord stimulation alone [36]. Similar effects have been reported for ankle joint kinematics: continuous spinal cord stimulation combined with rhythmic MG stimulation during the stance phase increased ankle joint excursion by approximately 4° [15]. In the present study, rhythmic L2 stimulation alone and combined L2+HAM+MG stimulation increased ankle joint excursion by approximately 6°. Under L2+HAM+MG stimulation, the increased ankle excursion was mediated by enhanced heel lift as well as an initial hallux lift occurring during the subsequent, unstimulated swing phase.

Analysis of heel and hallux kinematics revealed a characteristic sequence underlying forward propulsion. Propulsion begins with heel lift, while the hallux remains in contact with the treadmill belt. A slight deviation in the downward trajectory of the hallux indicates the onset of plantar flexion, corresponding to active push-off. Subsequently, both the heel and hallux lift simultaneously above the treadmill belt, marking the transition to the swing phase. In the control condition, the maximum velocity of heel movement from lift-off to peak elevation was 1041 ± 134 mm/s. This value remained essentially unchanged with L2 stimulation alone but increased significantly to 1253 ± 142 mm/s with combined L2+HAM+MG stimulation. Similarly, the maximum velocity of the hallux increased from 972 ± 68 mm/s in the control condition to 1150 ± 150 mm/s with L2+HAM+MG stimulation.

The temporal peaks of heel and hallux elevation coincided. Compared with control conditions, L2+HAM+MG stimulation increased heel lift amplitude by 42% (*p* < 0.001) and hallux lift amplitude by 30% (*p* < 0.001). Furthermore, the difference between heel and ankle elevation increased from 19 mm in the control condition to 25 mm during stimulation, indicating a stronger push-off from the support surface during spinal–muscular stimulation and, consequently, enhanced forward propulsion.

Foot dorsiflexion occurs primarily during the swing phase and is characterized by elevation of the limb above the treadmill belt accompanied by active hallux lift. During spinal–muscular stimulation, distinct features of foot dorsiflexion were observed. With T12 posterior root stimulation alone, ankle and heel lift heights remained unchanged. In contrast, combined T12+TA stimulation increased ankle lift relative to control values (from 69 ± 11 mm to 72 ± 12 mm). In addition, while the integral of heel displacement over the gait cycle remained unchanged during both T12 and T12+TA stimulation, the integral of hallux displacement increased significantly by 33% (*p* < 0.0001) during T12+TA stimulation (Figure 4b). During swing-phase stimulation with T12+TA, both heel and hallux trajectory lengths increased relative to control values (Figure 5).

In the control condition, the maximum velocity of heel movement from lift-off to peak elevation was 1061 ± 105 mm/s. This value increased to 1120 ± 107 mm/s with T12 stimulation and further to 1128 ± 109 mm/s with T12+TA stimulation, while heel lift height remained unchanged. During swing-phase T12+TA stimulation, the amplitude of the first peak of hallux lift changed only slightly. However, the amplitude of the second peak increased significantly to 53 ± 14 mm compared with 40 ± 13 mm in the control condition. In relative terms, the first peak increased by 11.82% (*p* < 0.01), and the second peak increased by 42% (*p* < 0.01) during T12+TA stimulation (Figure 5b). The increase in the second hallux lift peak is indicative of active foot dorsiflexion (Figure 5).

**Figure 6:**
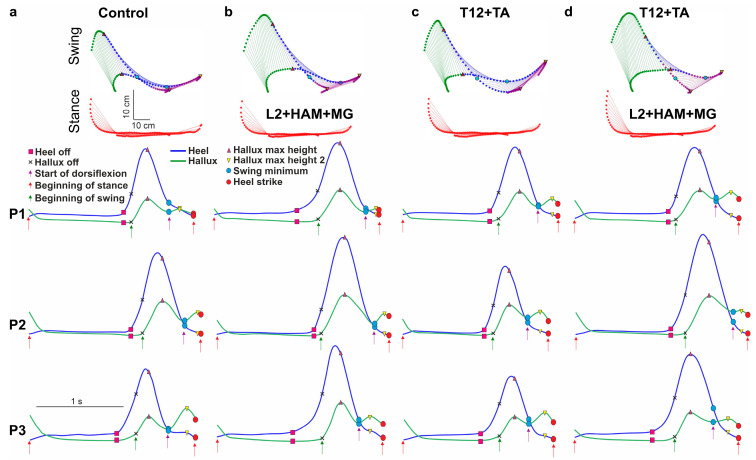
Stick diagrams and trajectories of heel and hallux movements during the stance and swing phases under control conditions (**a**), during stance-phase stimulation (L2+HAM+MG) (**b**), during swing-phase stimulation (T12+TA) (**c**), and during combined stance- and swing-phase stimulation (L2+HAM+MG+T12+TA) (**d**). Representative examples from three participants (P1, P2, P3) illustrate changes in heel and hallux trajectories under different stimulation conditions applied during specific phases of the gait cycle.

Forward propulsion and foot dorsiflexion were most effectively facilitated by combined stimulation consisting of L2+HAM+MG during the stance phase and T12+TA during the swing phase (Figure 6). Kinematic analysis revealed that heel and hallux movement patterns during L2+HAM+MG stimulation alone and during combined stimulation were largely similar across stance and swing phases, respectively. The high velocities of heel and hallux movement from lift-off to peak elevation during combined stimulation indicate highly effective propulsion. Under combined stimulation, heel lift height increased by 38% (*p* < 0.001), while the first and second hallux lift peaks increased by 31% (*p* < 0.001) and 19% (not significant), respectively. These findings provide strong evidence that combined stimulation of posterior roots and flexor/extensor muscles during the stance and swing phases enables effective control of both forward propulsion and foot dorsiflexion within a single gait cycle.

## 5. Limitations and Future Directions

Several limitations of the present study should be considered when interpreting the results and defining directions for future research. First, the experiments were conducted exclusively in healthy volunteers. Although this model enabled controlled investigation of the complementary interaction between spinal neuromodulation and peripheral muscle stimulation, the findings cannot be directly generalized to neurological populations. As discussed above, individuals with spinal cord injury or stroke may present altered excitability of spinal locomotor networks, impaired sensorimotor integration, and structural muscle changes, all of which may influence the effectiveness of phase-specific stimulation. Future studies should therefore evaluate this approach in clinical populations with gait impairments.

Second, gait modulation was examined only during treadmill walking. While this experimental setting allowed precise control of gait speed and stimulation timing, treadmill locomotion differs from overground walking in terms of sensory feedback, postural control, and propulsion mechanics. Given the potential clinical relevance emphasized in the Conclusions, future investigations should assess the proposed stimulation paradigm during overground walking and in more ecologically valid environments.

Third, phase-dependent stimulation was implemented using predefined temporal parameters rather than adaptive, real-time detection of gait events. Because effective interaction between spinal and peripheral inputs critically depends on accurate phase timing, future work should incorporate real-time biomechanical or electrophysiological feedback to optimize stimulation delivery and enhance inter-individual adaptability.

Fourth, the present study focused primarily on kinematic outcomes related to forward propulsion and foot dorsiflexion. Although these measures provide meaningful functional indicators, the proposed mechanisms involve modulation of spinal locomotor networks and motor neuron pools.

Future studies should integrate comprehensive electromyographic, reflex, and neurophysiological assessments together with detailed analyses of ground reaction forces to directly characterize neural and muscular activation patterns and to clarify the relative contributions of spinal and peripheral mechanisms.

Finally, the sample size was relatively small, which may limit statistical power and generalizability. Larger-scale studies are required to confirm the robustness of the observed effects and to explore inter-individual variability. Longitudinal studies are also warranted to determine whether repeated application of combined spinal and muscular stimulation can induce durable adaptations relevant for gait rehabilitation.

Together, addressing these limitations will be essential for translating the proposed multisegmental, phase-specific spinal and muscular stimulation approach into effective and clinically applicable rehabilitation strategies.

## 6. Conclusions

Loss of joint coordination and gait dysfunction are common consequences of stroke, and restoration of walking ability remains a primary goal of post-stroke rehabilitation. Conventional gait rehabilitation approaches include functional electrical stimulation (FES), robotic-assisted training, and brain–computer interfaces; however, each of these techniques has inherent limitations. For example, FES may enhance ankle dorsiflexion and reduce foot drop by activating dorsiflexor muscles, but weak plantar flexors can limit propulsion [37,38]. Moreover, only modest improvements in clinical and biomechanical gait parameters have been reported during early recovery following ischemic stroke [37]. Despite significant technological progress in the field of FES over the past 10–15 years, there is broad consensus that existing systems remain insufficiently advanced and require further development [39,40].

Cervical epidural stimulation of the spinal cord has been shown to increase cerebral blood flow and restore motor function [41,42]. Visocchi et al. reported enhanced regional cerebral perfusion during spinal stimulation in individuals with stroke, accompanied by improved voluntary movements, reduced spasticity, increased endurance, elimination of abnormal muscle co-activation, improved intermuscular coordination, and reduced clonus [42]. Nevertheless, improvements in motor performance may not be solely attributable to changes in cerebral hemodynamics [43].

Transcutaneous spinal cord stimulation has been proposed as a therapeutic adjunct to gait training, potentially enhancing rehabilitation outcomes in individuals with chronic stroke [9,44]. More recently, a novel gait modulation approach has been introduced that combines continuous stimulation of spinal locomotor networks with spatiotemporally specific activation of flexor and extensor motor pools during distinct phases of the gait cycle [15]. Although this approach can modulate gait parameters in stroke patients, it also exhibits certain limitations [45].

The results of the present and previous studies demonstrate that spinal neuromodulation combined with direct stimulation of leg muscles is an effective means of regulating stepping movements in healthy individuals. Targeted stimulation of posterior roots that activate flexor and extensor motor pools during specific phases of the gait cycle likely engages the spinal locomotor network composed of flexor and extensor half-centers. In parallel, direct stimulation of flexor and extensor muscles serves as a functional prosthesis by activating the appropriate muscles at specific times within the locomotor cycle. This dual mechanism dictates which muscles are activated and to what extent, thereby supporting the execution of stance and swing phases during stepping. Simultaneous engagement of these mechanisms produces complementary effects that enhance stepping performance.

Specifically, stimulation of posterior roots at L2 combined with activation of extensor muscles during the stance phase facilitates forward propulsion, whereas stimulation of posterior roots at T12 combined with activation of flexor muscles during the swing phase promotes effective foot dorsiflexion. Their combined application across stance and swing phases results in integration of spinal and peripheral inputs within the spinal locomotor network, enabling coordinated control of forward propulsion and foot dorsiflexion within a single gait cycle.

Spinal cord stimulation represents a promising therapeutic approach for gait impairments following cerebrovascular disorders, as it can modulate multiple levels of the central nervous system, including both spinal and supraspinal structures [46,47,48]. These findings underscore the need for the development of specialized rehabilitation protocols and advanced stimulation technologies, including multisegmental spinal neuromodulation strategies.

## Figures and Tables

**Table 1:** Study protocol.

Stage	Condition
1	walking without stimulation (control)
2	walking with L2 stimulation during stance phase
3	walking with L2+HAM stimulation during stance phase
4	walking with L2+HAM+MG stimulation during stance phase
5	walking with T12 stimulation during swing phase
6	walking with T12+TA stimulation during swing phase
7	walking with L2+HAM+MG stimulation during stance phase+T12+TA stimulation during swing phase

Abbreviations: L2, right spinal cord posterior roots at the L2 vertebral level; HAM, right hamstring muscles; MG, right gastrocnemius muscle; T12, right posterior roots at the T12 vertebral level; TA, right tibialis anterior muscle. The duration of each condition was 20–30 s.

## Data Availability

The data supporting the findings of this study are available upon request from the corresponding author.

## Supplementary Materials

The following supporting information can be downloaded at: https://www.mdpi.com/article/10.3390/life16020226/s1, Video S1: control walking without stimulation (top left), walking with stimulation of the right posterior root L2 and right extensor leg muscles during the stance phase of the right leg (bottom left), walking with stimulation of the right posterior root T12 and right flexor leg muscles during the swing phase of the right leg (top right), walking with stimulation of the right posterior root L2 and the right extensor leg muscles during the stance phase of the right leg, and stimulation of the right posterior root T12 and the right flexor leg muscles during the swing phase of the right leg (bottom right).

## Abbreviations

The following abbreviations are used in this manuscript:

AIS	ASIA Impairment Scale
ASIA	American Spinal Injury Association
FES	functional electrical stimulation
HAM	right hamstring muscles
L2	right spinal cord roots at L2 vertebrae level
MG	right m. gastrocnemius
SCI	spinal cord injury
T12	right spinal cord roots at T12 vertebrae level
TA	right m. tibialis anterior
TSV	tab-separated values
